# Meet Spinky: An Open-Source Spindle and K-Complex Detection Toolbox Validated on the Open-Access Montreal Archive of Sleep Studies (MASS)

**DOI:** 10.3389/fninf.2017.00015

**Published:** 2017-03-02

**Authors:** Tarek Lajnef, Christian O’Reilly, Etienne Combrisson, Sahbi Chaibi, Jean-Baptiste Eichenlaub, Perrine M. Ruby, Pierre-Emmanuel Aguera, Mounir Samet, Abdennaceur Kachouri, Sonia Frenette, Julie Carrier, Karim Jerbi

**Affiliations:** ^1^Psychology Department, University of MontrealMontreal, QC, Canada; ^2^Center for Advanced Research in Sleep Medicine, Hôpital du Sacré-Cœur de MontréalMontreal, QC, Canada; ^3^Blue Brain Project, École Polytechnique Fédérale de LausanneGeneve, Switzerland; ^4^Inter-University Laboratory of Human Movement Biology, University Claude Bernard Lyon 1Villeurbanne, France; ^5^DYCOG Lab, Lyon Neuroscience Research Center, INSERM U1028, UMR 5292, University Lyon ILyon, France; ^6^LETI Lab Sfax National Engineering School (ENIS), University of SfaxSfax, Tunisia; ^7^Department of Neurology, Massachusetts General Hospital (MGH), Harvard Medical SchoolBoston, MA, USA; ^8^Centre de Recherche de l’Institut Universitaire de Gériatrie de Montréal (CRIUGM)Montréal, QC, Canada; ^9^Centre de Recherche En Neuropsychologie Et Cognition (CERNEC), Psychology Department, Université de MontréalMontréal, QC, Canada; ^10^BRAMS, International Laboratory for Research on Brain, Music, and SoundMontreal, QC, Canada

**Keywords:** spindles, K-complex, automatic detection, sleep-EEG, spinky, open-source, toolbox, TQWT

## Abstract

Sleep spindles and K-complexes are among the most prominent micro-events observed in electroencephalographic (EEG) recordings during sleep. These EEG microstructures are thought to be hallmarks of sleep-related cognitive processes. Although tedious and time-consuming, their identification and quantification is important for sleep studies in both healthy subjects and patients with sleep disorders. Therefore, procedures for automatic detection of spindles and K-complexes could provide valuable assistance to researchers and clinicians in the field. Recently, we proposed a framework for joint spindle and K-complex detection (Lajnef et al., 2015a) based on a Tunable Q-factor Wavelet Transform (TQWT; Selesnick, 2011a) and morphological component analysis (MCA). Using a wide range of performance metrics, the present article provides critical validation and benchmarking of the proposed approach by applying it to open-access EEG data from the Montreal Archive of Sleep Studies (MASS; O’Reilly et al., 2014). Importantly, the obtained scores were compared to alternative methods that were previously tested on the same database. With respect to spindle detection, our method achieved higher performance than most of the alternative methods. This was corroborated with statistic tests that took into account both sensitivity and precision (i.e., Matthew’s coefficient of correlation (MCC), F1, Cohen κ). Our proposed method has been made available to the community via an open-source tool named Spinky (for spindle and K-complex detection). Thanks to a GUI implementation and access to Matlab and Python resources, Spinky is expected to contribute to an open-science approach that will enhance replicability and reliable comparisons of classifier performances for the detection of sleep EEG microstructure in both healthy and patient populations.

## Introduction

Ironically, a good night’s rest is often made possible by an active brain that exhibits complex macro and micro-structures of electrical activity at various spatial and temporal scales (Iber et al., 2007; Carskadon and Dement, 2011). Characteristic sleep stages are generally identified in 20 s or 30 s-long segments of physiological activity recorded with polysomnographic data, including prominently electroencephalographic signals (EEG). Sleep stages can be broadly split into four types: rapid-eye-movement (REM) and three non-REM (N1, N2, N3) (Rechtschaffen and Kales, 1968; Iber et al., 2007), and each stage is associated with specific cerebral signatures and functions. Furthermore, sleep EEG recordings contain characteristic micro-structures (i.e., short-lived stereotypical events) that are often considered to be hallmarks of sleep-related cognitive processes and, in some cases, a sign of sleep anomalies. Among these, K-complexes and sleep spindles are some of the most prominent micro-events that are studied in sleep studies. Given that they mainly occur during the N2 sleep stage, spindles and K-complexes not only guide experts during their scoring of sleep stages, but they are also thought to be key elements in the diagnosis of sleep disorders and the exploration of the functional role of sleep.

### Sleep Spindles

According to the American Academy of Sleep Medicine (AASM), sleep spindles are distinct EEG waves within the 11–16 Hz frequency range, they have a duration of ≥0.5 s, and they typically show a peak in amplitude over central brain regions (Iber et al., 2007). These waveforms have been shown to be generated by the thalamus—more specifically by the reticular nucleus, which acts as a pacemaker (Fuentealba and Steriade, 2005)—and they propagate to the cortex via thalamo-cortical projections (e.g., Steriade, 2003, 2005; Barthó et al., 2014; Lüthi, 2014). Over the last years, spindles have been the subject of many debates and a lot of research on the mechanisms and functions of the sleeping brain. Sleep spindles play an important role in memory consolidation during sleep (Schabus et al., 2004; Morin et al., 2008; Diekelmann et al., 2009; Diekelmann and Born, 2010; Barakat et al., 2011; Fogel et al., 2012; Lafortune et al., 2014) and they undergo age-related changes (e.g., Seeck-Hirschner et al., 2012; Martin et al., 2013). Consequently, alterations in spindle density (number per minute) can be a symptom of neurological disorders such as dementia (e.g., Ktonas and Ventouras, 2014; Latreille et al., 2015), schizophrenia (e.g., Ferrarelli et al., 2010; Ferrarelli and Tononi, 2011), depression (Riemann et al., 2001), REM sleep behavior disorder (O’Reilly et al., 2015), Parkinson’s disease (Christensen et al., 2015; Latreille et al., 2015), stroke recovery, mental retardation and sleep disorders (De Gennaro and Ferrara, 2003).

### K-Complexes

K-complexes are well delineated negative sharp waves that are immediately followed by a positive component. Their total duration is of ≥0.5 s, and they typically peak in amplitude over frontal electrodes (Iber et al., 2007). The role of K-complexes in sleep is however still a matter of debate. Since they are often followed by micro-awakenings (Halász, 2005), they are often considered to be an arousal response. Moreover, some studies suggest that K-complexes have a sleep “protection” function (Jahnke et al., 2012). Lastly, single-unit recordings during human sleep have suggested that K-complexes may represent isolated down-states (Cash et al., 2009).

### Spindle and K-Complex Detection

A reliable detection of sleep spindles and K-complexes in EEG recordings is of major importance in numerous basic and clinical sleep investigations. Visual annotation of sleep spindles and K-complexes is tedious, time consuming, subjective and prone to human errors. As a consequence, the inter-rater agreement for visual spindles and K-complexes scoring reported in the literature is remarkably low (Zygierewicz et al., 1999; Devuyst et al., 2010; Warby et al., 2014). Therefore, just like in sleep staging (e.g., Lajnef et al., 2015b), automatic or semi-automatic procedures are expected to be of great utility for the detection of sleep spindles and K-complexes. Straightforward approaches based on band-pass filtering and thresholding have been proposed for both spindles and K-complexes detection (e.g., Huupponen et al., 2000; Devuyst et al., 2010). Other techniques that have been proposed include template-based filtering, using matching pursuit (e.g., Schönwald et al., 2006), filtering approaches based on continuous wavelet transforms (Erdamar et al., 2012) and signal classification methods based on artificial neural networks (ANN; e.g., Günes et al., 2011), Support Vector Machines (SVMs; e.g., Acir and Güzeliş, 2004) or decision-trees (Duman et al., 2009). However, few have investigated the detection of K-complexes and spindles simultaneously using a common methodological framework (Jobert et al., 1992; Koley and Dey, 2012; Jaleel et al., 2013; Camilleri et al., 2014; Lajnef et al., 2015a; Parekh et al., 2015).

### Goal of This Study

In a recent study, we proposed a framework for joint spindle and K-complex detection, based on the combination of a discrete wavelet transform, known as the Tunable Q-factor Wavelet Transform (TQWT; Selesnick and Bayram, 2009; Selesnick, 2011a,b,c) and morphological component analysis (MCA). Appropriate Q-factor tuning allows for the decomposition of the EEG signal into transient (K-complex) and oscillatory (spindle) components. Our results from the sleep EEG recordings of 14 participants demonstrated that this framework could be a promising tool to facilitate and improve the reliability of the detection of spindles and K-complexes. This study extends our previous work in three significant ways. First, we provide critical validation and benchmarking of the TQWT-MCA approach by applying it to an open-access database, namely the Montreal Archive of Sleep Studies (MASS; O’Reilly et al., 2014). Second, we extend on the performance measures by including a wide range of metrics (sensitivity, positive predictive value (PPV), Matthew’s coefficient of correlation (MCC), Cohen’s κ and the F1 measure). This is important for the comparisons with other methods in the field. Finally, with the publication of this report we provide an open-source version of the software (which we coined Spinky for automatic spindle and K-complex detection), and we describe all the processing steps necessary for users to test on their data or replicate our findings.

### Article Outline

The article is organized as follows. We first describe the open-access database that we used (“Databases” Section). Next, in Sections “Optimal Threshold Estimation and Detection”, we provide a thorough investigation of the threshold estimation step (training phase of our algorithm), followed by a presentation of the statistical assessment of detection results (“Statistical Assessment of Detection” Section). Section “Performance Evaluation and Comparison with other Algorithms” provides the links to the open-access Matlab-based toolbox and associated Python (Jupyter) interactive notebook. In “Open Access” Sections, a user-oriented overview of the Matlab GUI software is overviewed. The results section provides an assessment of the robustness of the threshold estimation step (“Evaluation of the Detection Threshold Variability” Section), followed by the results of the automatic detection of spindles and K-complexes (“Automatic Spindle and K-Complex Scoring with Spinky” Section). Finally, in Sections “Discussion”, we discuss our results and future work.

## Materials and Methods

### Databases

To demonstrate the performance of the proposed detector and facilitate comparisons with other methods, we chose to examine its detection results on an open-access database: MASS (O’Reilly et al., 2014). More specifically, we used the second subset of the first cohort (C1/SS2). This contained 19 full night recordings of healthy young participants, all scored for spindles and K-complexes by experts. Scoring was performed on N2 epochs using the C3 derivation and a linked-ear reference. As discussed in O’Reilly and Nielsen (2015), the two experts for sleep spindles show relatively low inter-rater agreement (a median Cohen κ of about 0.4) owing to the fact that the first one scored spindles using traditional AASM rules, whereas the second rater used an approach similar to the one employed in Ray et al. (2010)^1^. It is also worth noting that the second expert only scored 15 out of the 19 nights. Moreover, scoring of K-complexes was performed with a minimal duration of 0.5 s and a minimal peak-to-peak amplitude of 75 uV. The experts did not score K-complexes during short-period N2 intrusions in REM sleep.

### Optimal Threshold Estimation and Detection

As described in Lajnef et al. (2015a), the TQWT-MCA approach requires an initial training/calibration step, where a small subset of the EEG data is visually scored for spindles or K-complexes, and then used to derive an optimal detection threshold. Thus, the operating point for the detection trade-off between type I and type II classification errors depends on this *a priori* specification of a detection threshold. The best value for such a threshold is subject-dependent and can best be determined by assessing it on a small subset of expert scorings (i.e., by *training* the detector on this subset). To complement the analyses reported in Lajnef et al. (2015a) and to improve our understanding of the parameters affecting the choice of optimal detection conditions, we performed two sets of trainings:

- *Analysis 1*: A random selection of 10 minutes of scoring (i.e., 30 scoring pages of 20 s) was used to compute the optimal detection threshold corresponding to each expert scoring. This process was repeated 10 times and the distribution of these thresholds was then estimated. The first, second and third quartiles of these distributions were entered as parameters for the detections associated with each scoring.
- *Analysis 2*: We randomly chose a number N of 20 s scoring pages. This N number was itself randomly drawn from a uniform distribution spanning values from 15 to 120. This process was repeated 60 times per expert scoring to evaluate the impact of the number of scoring pages (i.e., of N) on the variability of the estimated threshold, and also to improve on the optimal number of pages that should be scored by experts for a reliable automatic scoring of the remaining pages. Thresholds determined on samples associated with N within the 15–120 range were separated in six bins of equal width, each containing an average of 10 samples per expert scoring. Then, the mean and the standard deviation (SD) of these thresholds were computed per scoring per bin. Standard least-square regressions were computed to evaluate the impact of N on the expected value of the threshold and on the reliability of the threshold estimation. For spindles, an additional linear factor was added to these regression models to take into account the impact of the expert (i.e., choosing scoring by expert 1 or expert 2 as ground truth). No interaction term between N and the expert was used as it was not statistically significant (*p* = 1.00 for means, *p* = 0.65 for SD).

### Statistical Assessment of Detection

Detection performances were assessed using a sample-based computation of the following statistics: sensitivity, PPV, MCC, Cohen’s κ and the F1 measure. Details of this statistical approach can be found in O’Reilly and Nielsen (2015). With respect to K-complexes, the detector reported only on the position, and not the duration, of the negative peak. Thus, for both the expert and the detector scoring, the duration of a detected K-complex was defined as the time window starting 0.1 s before and ending 1.3 s after its negative peak.

### Performance Evaluation and Comparison with Other Algorithms

An important advantage of using an open-access database is the ability to benchmark the performance of a new algorithm and compare it to other methods. After running Spinky on the MASS data sets, we compared its performance to those of other spindle detection algorithms published in the literature, including a Teager detector (Ahmed et al., 2009), Sigma index (Huupponen et al., 2000, 2007), RSP (Devuyst et al., 2011), RMS (Mölle et al., 2002; all four assessed in O’Reilly and Nielsen, 2015), as well as a detector based on matching pursuit (MP; Durka et al., 2015). The output of the different methods was compared using the five metrics described in the previous section (Sensitivity, PPV, MCC, Cohen’s κ and F1). These comparisons were expected to be highly reliable as the same assessment method, subject sample and expert scoring were used for all cases.

We further compared our results with detectors that were applied on the same database, but using slightly different methods (e.g., TP, TN, FP and FN determined on time windows instead of time samples) or using a sub-sample of subjects (e.g., excluding, for the computation of test statistics, subjects that were used for training the detector). This second set included the eight detectors A1–A8 that were tested in Tsanas and Clifford (2015), a detector based on complex demodulation (CD; Ray et al., 2015) and two detectors using random forest (RF) and ANN that were assessed in Patti et al. (2015).

### Open Access

To allow others to replicate our results or use our method on their own data, we provide open-access code, GUIs and interactive resources for the developed tools. The code used for assessing the performance of the detector is available as a Jupyter (IPython) notebook at https://github.com/christian-oreilly/spinkyDemo/blob/master/notebook/finalDemo.ipynb. The database used for spindle detection is freely available at http://www.ceams-carsm.ca/en/MASS. The MATLAB (Mathworks Inc., MA, USA) source code and GUIs for the detector can be downloaded at https://github.com/TarekLaj/SPINKY.

### Spinky: A Matlab GUI Interface for Spindles and K-Complex Detection

**Figure 1:**
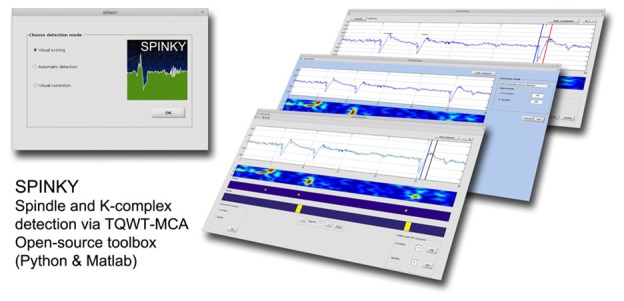
**Overview of the Spinky toolbox.** Left: the main GUI used to launch the required module. Right: snapshots of the three main modules available with Spinky.

The spindle and K-complex detection pipeline used in this article has been implemented in a freely available toolbox called Spinky. The MATLAB package contains three modules allowing for: (i) visual K-complex and spindle scoring (on a subset of data for training purposes); (ii) automatic detection; and (iii) manual correction of false detections. In principle, the output of the first module is used in the automatic detection, and the result of this module is the input to the third module (Figure 1). Once installed, the toolbox can be launched by typing “spinky” in the MATLAB command window. This will activate the main toolbox window (Figure 1—Left), and allows to launch one of the three main modules. The basic principles of how to use each module is described below.

#### STEP 1: Visual Detection

**Figure 2:**
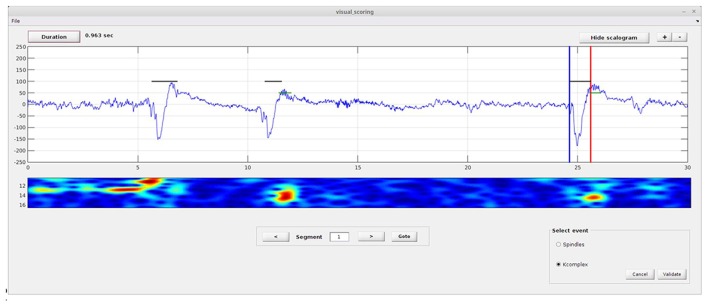
**Spinky visual scoring module.** Illustration of the module provided for visual scoring of K-complex and spindle events. The interface includes a select event panel (spindles or K-complexes), and buttons to validate or cancel the scoring. Buttons for duration estimation, zoom and display of the scalogram are also provided. A larger version of this figure is provided online (https://raw.githubusercontent.com/TarekLaj/SPINKY/master/Screenshots/Figure_2_Lajnef_et_al_frontneuroinf_w2017.jpg).

This module (Figure 2) allows the user to manually mark spindle and K-complex events on a single EEG channel. In principle, this only needs to be done on a small sample of data, as it will subsequently be used for training. To perform the visual scoring the user needs to go through the following steps. First, the beginning and end of the event to score must be marked by moving the blue (begin) and red (end) lines using the mouse (drag and drop). Next, the user selects the event type using radio buttons (on “Select event” panel). Clicking the “Validate” button saves the results, and generates a text file named “scorer_name_subject name_kcomplex.txt” and/or “scorer_name_subject name_spindles.txt”. The user can then move to the next/previous segment using the next/previous arrow buttons, or move directly to a desired segment by entering the segment number in the text box and clicking the “goto” button.

Note that if the desired detection threshold is already known, or if the user wants to manually choose and experiment with some threshold values, they can skip this visual scoring step and start directly with the automatic detection module.

#### STEP 2: Automatic Detection

**Figure 3:**
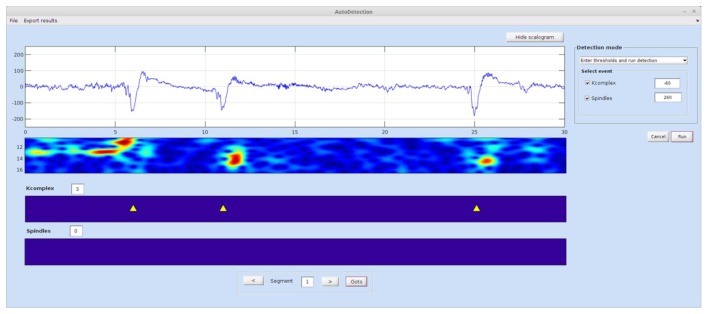
**Spinky automatic detection module.** The detection mode panel allows the user to switch between two types of operations: manual entry of a threshold for each event type, and deriving the optimal threshold via the ROC approach based on training samples of data. Detected events are shown in the panels at the bottom of the interface. The time-frequency map can be hidden using the show/hide scalogram button. An “Export Results” menu at the top of the GUI allows the user to export detection statistics. A larger version of this figure is provided online (https://raw.githubusercontent.com/TarekLaj/SPINKY/master/Screenshots/Figure_3_Lajnef_et%20_al_frontneuroinf_2017.jpg).

This module (Figure 3) runs an automatic detection of spindles and K-complexes using the TQWT-MCA method (Lajnef et al., 2015a). To run this module the user must first load the single-channel EEG data and select the required detection mode from the menu list.

##### Case 1

If the threshold value is already known (i.e., based on previous training or the user wishes to set it manually), the user can choose “Enter thresholds and run detection” mode. This will launch the automatic detection, while skipping the training step. Example values for the K-complex and spindle values are −60 and 200, but these values can change depending on the data.

##### Case 2

To determine the best threshold value using a visually scored data sample, the user must select “Compute thresholds and run detection”. The user will be asked to choose the training EEG data files and associated visual scoring files. In such a case, the visual scoring file should either be the output of the Visual Scoring module, or a text file in the exact same format (see Appendix A in the Spinky online manual for details).

Once the detection is complete and the results are saved to disk, the user can compute statistics on the detected events for the whole data sets (all epochs), by clicking on the menu button “Export results”. This function will compute statistics and save them to a .txt or .mat file. The statistics that are currently available for K-complex events are: total number, density, frequency and mean amplitude. For spindles, the available statistics include: total number, density, mean duration, frequency and mean amplitude.

#### STEP 3: Manual Correction of False Detections (Optional)

**Figure 4:**
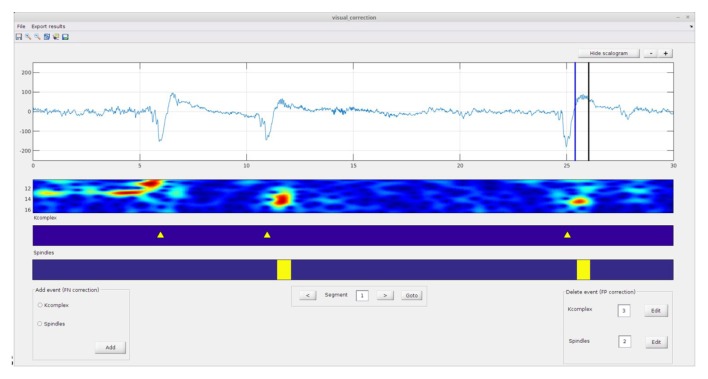
**Spinky visual correction module: the results of the automatic detection module (previous step—Figure 3) can be manually corrected here.** Adding or removing events are both supported (see left and right corner panels). A larger version of this figure is provided online (https://raw.githubusercontent.com/TarekLaj/SPINKY/master/Screenshots/Figure_4_Lajnef_et_al_frontneuroinf_2017.jpg).

This module allows the user to manually correct the output of the automatic detection procedure. The visual correction interface (Figure 4) allows correcting for both false negatives (i.e., to manually add an event that the automatic detector missed) and false positives (i.e., to delete events detected by the algorithm, but considered to be false detections by the user). Briefly, to carry out these corrections the user must first load the automatic score files (i.e., the .txt file generated by automatic detection module). In order to delete a false positive event, the user must click the edit button, select the event to remove and press the delete button. To add a spindle or K-complex event missed by the automatic detector, the user must select the event type on the “add event panel” and then move the selection line(s) to the desired position(s) and press the “add” button. Note that it is also possible to continue work on a previously initiated session; in this case corrections will append the existing corrected text file.

## Results

### Evaluation of the Detection Threshold Variability

#### First Analysis

**Figure 5:**
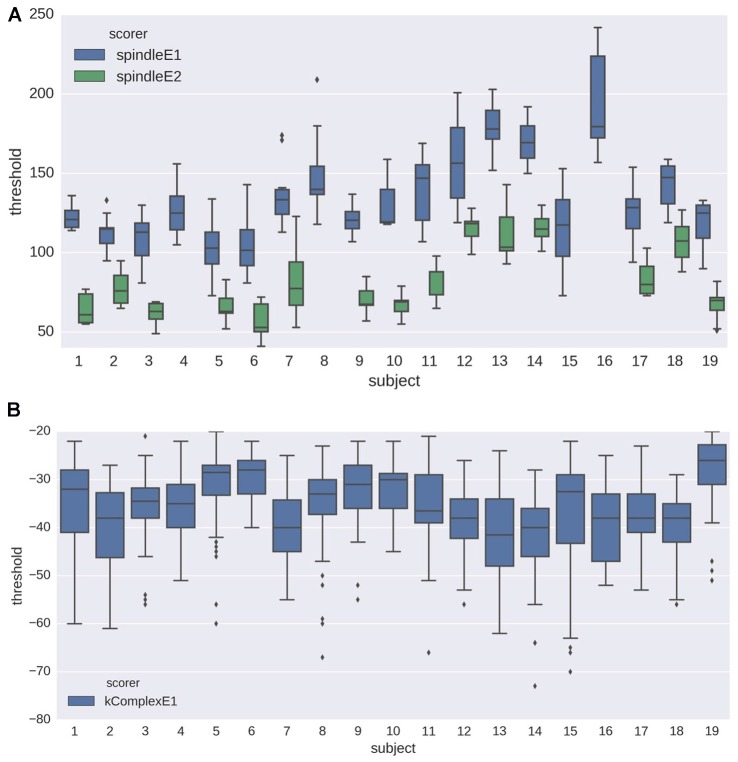
**Box plots showing the distribution of the detection thresholds estimated from the training step (see analysis 1 in Section “Optimal Threshold Estimation and Detection” for details) for (A)** spindles and** (B)** K-complexes. The results are depicted for data from each subject, using both experts as gold standard (only one expert annotation was available for K-complex scoring).

The initial *training step* of the proposed pipeline estimates the optimal detection threshold based on a sample of annotated EEG data. As explained in “Optimal Threshold Estimation and Detection” Sections, we evaluated the robustness of this training phase with two analyses. Figure 5 shows the distribution of estimated detection thresholds obtained in the first analysis (i.e., *N* = 30; 10 random draws) for spindles and K-complexes. For spindles (Figure 5A), the impact of the subject and the expert, on both the mean and the SD of the estimated thresholds, are clearly visible. For K-complexes, we also observe a fair amount of inter-subject variability (central tendency and spread), which however appears to be less noticeable than for spindles.

#### Second Analysis

**Figure 6:**
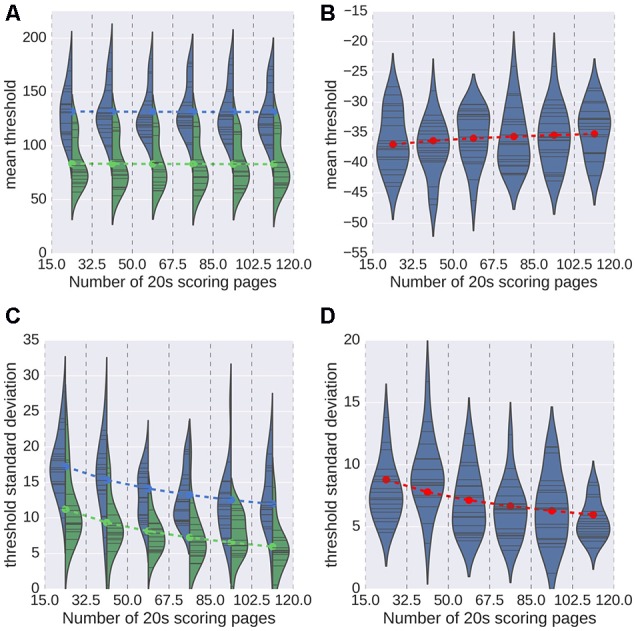
**Mean value (A,B)** and standard deviation (SD; **C,D**) of the estimated threshold for every subject (indicated as thin black bars within the plotted distributions) for spindles **(A,C)** and K-complexes **(B,D)**. In the case of spindles, results for each expert is reported (color coded blue and green). Dashed lines indicate the optimal value predicted by a linear regression that considered the value of N and, for spindles, the expert.

The results obtained for the second analysis are displayed in Figure 6. As a reminder, for this analysis, we randomly chose a number N of 20 s scoring pages, with N drawn from a uniform distribution spanning values from 15 to 120. As can be seen in Figures 6A,B, the number of pages used does not have a significant impact on the mean detection threshold (ordinary least-square regression *t* = −0.10, *p* = 0.92 for spindles; *t* = 1.32, *p* = 0.19 for K-complexes). This finding indicates that using a smaller number of pages for training induces no bias in the estimation of the optimal detection threshold. The impact of the number of pages on the SD of the estimated thresholds is shown in Figures 6C,D. Dashed lines were overlaid to these violin plots to indicate the threshold value predicted by the linear model, linking the threshold to the experts and the logarithm (base 2) of the number N of scoring pages. With respect to the SD, this model captures almost half of the observed variance for spindles (*R*^2^ = 0.419), and about an eighth for K-complexes (0.123). According to this model, the expected deviation from the mean value (i.e., the optimal threshold) will decrease by approximately 2.4 every time we double the number of scoring pages used during training. Globally, both the distribution of mean and the SD values confirm that a small number of 20 s scoring pages is sufficient to achieve a stable estimation of the detection threshold.

### Automatic Spindle and K-Complex Scoring with Spinky

**Figure 7:**
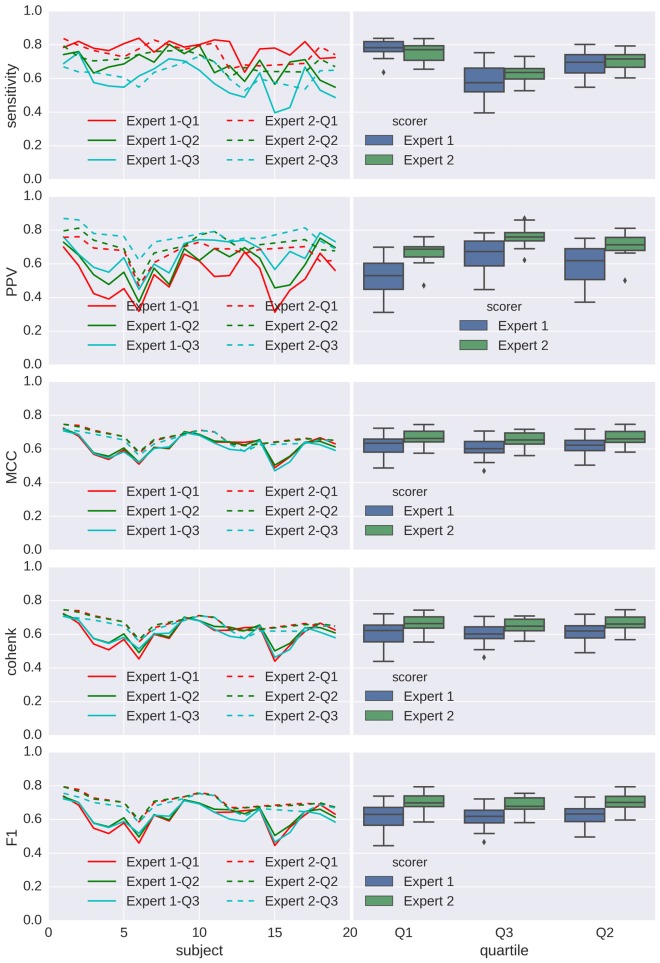
**Applying Spinky for spindle detection performance (Tunable Q-Factor Wavelet Transform (TQWT)-morphological component analysis (MCA) method) to sleep data from the open-access Montreal Archive of Sleep Studies (MASS) database.** Left column: principal statistics (sensitivity, positive predictive value (PPV), MCC, Cohen’s κ and F1) characterizing the performance of the detector when compared with scoring from experts (different types of line for different experts), for every subject (in *x* axis) and using a detection threshold taken as the first, second and third quartile (color coded) of the threshold distribution for the first analysis (*N* = 30). Right column: box-plots associated to each expert/quartile conditions.

**Figure 8:**
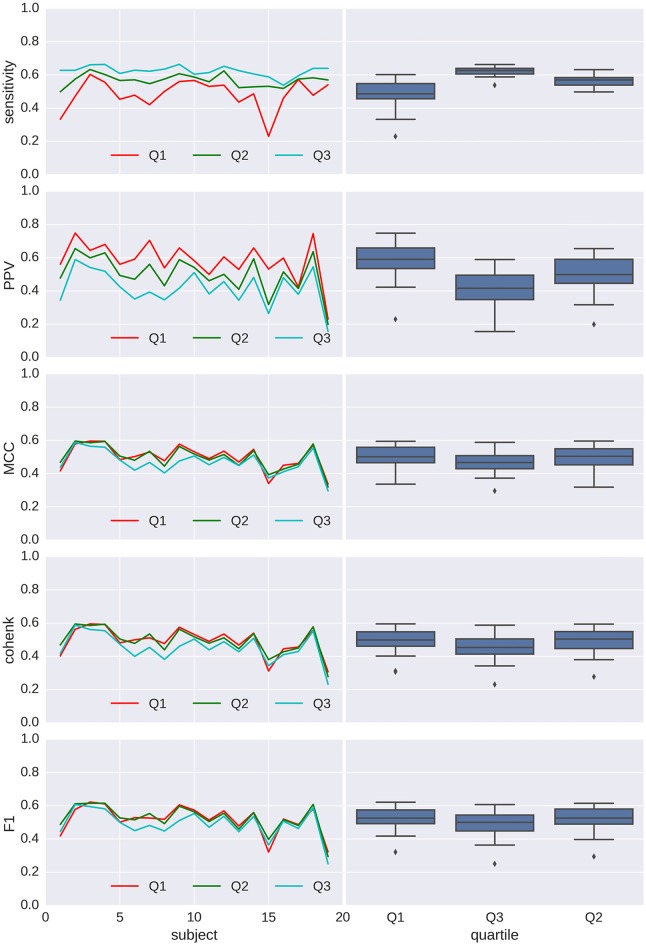
**Applying Spinky for K-complex detection performance (TQWT-MCA method) to sleep data from the open-access MASS database.** Left column: main statistics (sensitivity, pPV, Matthew’s coefficient of correlation (MCC), Cohen’s κ and F1) characterizing the performance of the detector when compared to scoring from experts (different type of line for each experts), for every subject (in *x* axis) and using a detection threshold taken as the first, second and third quartile (color coded) of the threshold distribution for the first analysis (*N* = 30). Right column: box-plots associated with each quartile conditions.

Performances for detection are shown in Figures 7, 8 for spindles and K-complexes, respectively. The average interquartile range of the distribution of estimated thresholds is of 19.1 for spindles and 11.7 for K-complexes. Thus, differences between computed statistics for the first quartile (box-plots on the left) and the third quartile (box-plots on the right) are typical of what can be observed with an imprecision of roughly ±10 around the mean in the estimation of the detection threshold for spindles and ±6 for K-complexes.

### Benchmarking the Performance of Spinky on MASS

In addition to evaluating the performance of our method as a function of its own parameters (previous sections), we also compared its performance with other detectors that have been reported in the literature. In this study, this was made possible by applying our method to open-access annotated sleep EEG recordings (O’Reilly et al., 2014), which were previously used to evaluate other detectors. Our proposed method performs favorably compared to the other approaches in terms of statistics, capturing both sensitivity and precision (MCC, Cohen κ and F1). The only exception was a superior result of CD on F1, for the first expert only (see Table 1).

**Table 1:** **Benchmarking spindle detection with Spinky**.

	Sensitivity		PPV		MCC		Cohen κ		F1	
	Exp1	Exp2	Exp1	Exp2	Exp1	Exp2	Exp1	Exp2	Exp1	Exp2
RSP	0.60	0.30	0.61	0.82	0.60	0.50	0.61	0.40	0.60	0.42
RMS	0.60	0.38	0.56	0.81	0.58	0.55	0.58	0.42	0.50	0.60
Sigma	0.62	0.29	0.60	0.81	0.60	0.56	0.60	0.48	0.61	0.56
Teager	0.63	0.26	0.58	0.85	0.58	0.50	0.59	0.43	0.60	0.44
MP	0.63	−	0.47	−	0.52	−	0.49	−	0.54	−
TQWT	**0.78**	**0.77**	**0.53**	**0.69**	**0.64**	**0.66**	**0.62**	**0.66**	**0.63**	**0.70**
A1	0.66	−	0.17	−	−	−	0.20	−	−	−
A2	0.17	−	−	−	−	−	0.22	−	−	−
A3	0.74	−	0.25	−	−	−	0.28	−	−	−
A4	0.66	−	0.52	−	−	−	0.51	−	−	−
A5	0.41	−	0.55	−	−	−	0.38	−	−	−
A6	0.73	−	0.31	−	−	−	0.37	−	−	−
A7	0.94	−	0.17	−	−	−	0.24	−	−	−
A8	0.77	−	0.14	−	−	−	0.16	−	−	−
CD	0.69	0.75	0.73	0.36	−	−	−	−	0.71	0.49
RF	−	0.71	−	0.53	−	−	−	−	−	−
ANN	−	0.68	−	0.55	−	−	−	−	−	−

*Summary of results of performance metrics (Sensitivity, positive predictive value (PPV), Matthew’s coefficient of correlation (MCC), Cohen κ and F1) computed for spindle detection in the Montreal Archive of Sleep Studies (MASS) database using Spinky and various other methods. Values in bold represent the performances of our TQWT based detection method (implemented in spinky)*.

## Discussion

The Spinky toolbox described and evaluated in this article appears to be a robust, efficient and convenient framework for joint spindle and K-complex detection. By combining a discrete wavelet transform known as the TQWT (Selesnick, 2011a) with MCA, Spinky allows for the decomposition of the EEG signal into transient (K-complex) and oscillatory (spindle) components (Lajnef et al., 2015a).

Selecting an appropriate detection threshold is a key step in the proposed method. Although this parameter can be set manually in Spinky, the recommended procedure is to use sample data (with visually scored events) to determine the optimal threshold using ROC analyses. Ideally, the small sample data to score for training should emanate from N2 epochs, as this is the stage where the targeted events are most prominent. But in theory, using epochs from other stages for training is possible too. In fact, in a previous study, we examined the effect of using N2 vs. other sleep stages for threshold selection using the same detection method (Lajnef et al., 2015a). In that study we used two scenarios, one where the training was uniquely done on N2 segments, and another one, where we used a balanced mixture of N2 and non-N2. Interestingly, our results showed that, given sufficient epochs, the training worked equally well in both scenarios. This indicates that the training does not need to occur only on N2 epochs. However, of course, one needs to ensure that spindle or K-complex events are available in the epochs used for training.

Furthermore, when analyzing the effect of different numbers of scored 20 s pages on detection threshold mean and variance, we found that 10 min (i.e., 30 pages of 20 s) seems to be sufficient. In addition, we also tested the effect of using a “minimal number of spindles” approach vs. a “minimal number of pages” approach on the robustness of the identified threshold. With a model “threshold ~ log_2_(pages) + scorer” we obtained *R*^2^ = 0.419 (i.e., we explained 41.9% of the variance in our data). Alternatively, using the number of spindles with a model “threshold ~ log_2_(spindles) + scorer”, we obtained *R*^2^ = 0.423. This high similarity between the two values of *R*^2^ indicates that we can interchangeably use the number of spindles or number of epochs to characterize the duration of the training.

The current study builds upon, and extends, our previous work in several significant ways. First, we provide critical validation and benchmarking of our TQWT-MCA approach by applying it to an open-access database, namely the MASS (O’Reilly et al., 2014). The automatic detection results reported here confirm the high detection performances we had previously obtained with this method in a different set of subjects (Lajnef et al., 2015a). Furthermore, the scores obtained in the current study were compared to those previously reported for other methods tested on the same database. For spindle detection, our method provided higher, or at least similar, performance on all statistics, taking into account both sensitivity and precision (i.e., MCC, F1, Cohen κ). As shown in Table 1, the sensitivity of spindle detection with Spinky was substantially higher than that of all the other methods (for both experts), except for the A3, A7 and A8 detectors, which obtain good sensitivity only by accepting a much lowered precision. No such benchmarking was possible for K-complex detection, since—to the best of our knowledge—no open-access tools for k-complex tools are available. A further important contribution of this study is the extensive evaluation of performance that was conducted using a wide range of metrics (sensitivity, PPV, MCC, Cohen’s κ and the F1 measure).

Importantly, with the publication of this report, we provide open-source Matlab code of our Spinky toolbox, along with Python-based interactive resources. The “Materials and Methods” Section of the present article, the step-by-step procedure, and the Matlab GUIs, that will hopefully allow other researchers, clinicians and students to use Spinky on their own data.

The thorough testing that we conducted on the training part of our pipeline (i.e., estimation of the best detection threshold based on annotated data samples) confirms the efficiency of the global framework. Indeed, the TQWT-MCA procedure implemented in Spinky would have been of limited value if copious amounts of visually scored pages were required for the training phase, as this would defeat the point of an automatic detector. Fortunately, our results suggest that there is no need to invest much resources in scoring a large number of pages before using these detectors. For example, for spindle detection, the SD of the optimal (i.e., the mean) value of the estimated threshold will drop by about 2.4 every time the N number of scoring pages is doubled. Moreover, the inter-rater agreement between experts and Spinky (MCC, Cohen’s κ, or F1) only slightly changed over the tested interquartile range of 19.2. In practice, our evaluation indicates that visual scoring of about 10 min per night seems sufficient. For K-complex detection, the SD of the thresholds dropped by about 1.3 every time the N number is doubled. In this case, the lower end of the threshold distribution seems to provide better inter-rater agreement.

To the best of our knowledge, Spinky is the first open-source tool that has been thoroughly evaluated for the detection of both spindles and K-complexes.

An alternative pragmatic approach to determining the best detection threshold, aside from systematically training Spinky on sample data, would be to run the automatic detector with a relatively low threshold, and then to correct the output manually using Spinky’s visual correction interface. This semi-automatic detection framework would yield high sensitivity by ensuring that all or most events are detected in the automatic detection module, as well as low false positive rates by manually discarding false detections in the visual correction module. Although this comes at the expense of more time for the manual correction, it could be a powerful technique, in particular when working with data with poor signal-to-noise ratio or with data sets collected across multiple centers using different EEG acquisition systems and settings.

In summary, the proposed spindle and K-complex detection framework provides robust performance with reasonably low time investment from the user. With the validation of our method on the open-access MASS sleep database, and the release of Spinky as an open-source tool (GUIs and Matlab/Python scripts), we sincerely hope that this work will be useful to the scientific and clinical community.

## Ethics Statement

All subjects gave written informed consent for their participation in the experiment during which their polysomnographic signals were recorded, in accordance with the Declaration of Helsinki. Pooling of the anonymized recording necessary for the creation of MASS was approved by the Comité d’éthique de la Recherche du Centre de Recherche de l’Hôpital du Sacré-Coeur de Montréal (Project Ref 2013-935; BQ-935).

## Author Contributions

TL, COR, MS, AK, JC and KJ wrote the article and designed the study. TL developed the Matlab code for script and GUI versions of Spinky. COR evaluated method performance and wrote the python notebook. SC, EC and P-EA actively contributed to software and GUI development. PMR, J-BE and SF provided visual scoring and helped with software testing. KJ supervised the design of the software and the validation procedure.

## Conflict of Interest Statement

The authors declare that the research was conducted in the absence of any commercial or financial relationships that could be construed as a potential conflict of interest.

## Footnotes

^1^Broad-band EEG signals (0.35–35 Hz band) and sigma filtered signals (11–17 Hz band) were both used in scoring to facilitate the identification of short duration, small amplitude or obscured (e.g., by delta waves or K-complexes) spindles. Thus, this second scoring is less “conservative” in the sense that they report higher spindle density.

## References

[B1] Acir N. Güzeliş C. (2004). Automatic spike detection in EEG by a two-stage procedure based on support vector machines. Comput. Biol. Med. 34, 561–575. 10.1016/j.compbiomed.2003.08.003 15369708

[B2] Ahmed B. Redissi A. Tafreshi R. (2009). An automatic sleep spindle detector based on wavelets and the teager energy operator. Conf. Proc. IEEE Eng. Med. Biol. Soc. 2009, 2596–2599. 10.1109/IEMBS.2009.5335331 19965220

[B3] Barakat M. Doyon J. Debas K. Vandewalle G. Morin A. Poirier G.. (2011). Fast and slow spindle involvement in the consolidation of a new motor sequence. Behav. Brain Res. 217, 117–121. 10.1016/j.bbr.2010.10.019 20974183

[B4] Barthó P. Slézia A. Mátyás F. Faradzs-Zade L. Ulbert I. Harris K. D.. (2014). Ongoing network state controls the length of sleep spindles via inhibitory activity. Neuron 82, 1367–1379. 10.1016/j.neuron.2014.04.046 24945776 PMC4064116

[B5] Camilleri T. A. Camilleri K. P. Fabri S. G. (2014). Automatic detection of spindles and K-complexes in sleep EEG using switching multiple models. Biomed. Signal Process. Control 10, 117–127. 10.1016/j.bspc.2014.01.010

[B6] Carskadon M. A. Dement W. C. (2011). “Normal human sleep: an overview,” in Principles and Practice of Sleep Medicine, 5th Edn. eds Kryger M. H. Roth T. Dement W. C. (St. Louis: Elsevier Saunders), 16–26.

[B7] Cash S. S. Halgren E. Dehghani N. Rossetti A. O. Thesen T. Wang C.. (2009). The human K-complex represents an isolated cortical down-state. Science 324, 1084–1087. 10.1126/science.1169626 19461004 PMC3715654

[B8] Christensen J. A. E. Nikolic M. Warby S. C. Koch H. Zoetmulder M. Frandsen R.. (2015). Sleep spindle alterations in patients with Parkinson’s disease. Front. Hum. Neurosci. 9:233. 10.3389/fnhum.2015.00233 25983685 PMC4416460

[B9] De Gennaro L. Ferrara M. (2003). Sleep spindles: an overview. Sleep Med. Rev. 7, 423–440. 10.1053/smrv.2002.0252 14573378

[B10] Devuyst S. Dutoit T. Stenuit P. Kerkhofs M. (2010). Automatic K-complexes detection in sleep EEG recordings using likelihood thresholds. Conf. Proc. IEEE Eng. Med. Biol. Soc. 2010, 4658–4661. 10.1109/IEMBS.2010.5626447 21096240

[B11] Devuyst S. Dutoit T. Stenuit P. Kerkhofs M. (2011). Automatic sleep spindles detection—overview and development of a standard proposal assessment method. Conf. Proc. IEEE Eng. Med. Biol. Soc. 2011, 1713–1716. 10.1109/IEMBS.2011.6090491 22254656

[B13] Diekelmann S. Born J. (2010). The memory function of sleep. Nat. Rev. Neurosci. 11, 114–126. 10.1038/nrn2762 20046194

[B12] Diekelmann S. Wilhelm I. Born J. (2009). The whats and whens of sleep-dependent memory consolidation. Sleep Med. Rev. 13, 309–321. 10.1016/j.smrv.2008.08.002 19251443

[B14] Duman F. Erdamar A. Eroğul O. Telatar Z. Yetkin S. (2009). Efficient sleep spindle detection algorithm with decision tree. Expert Syst. Appl. 36, 9980–9985. 10.1016/j.eswa.2009.01.061

[B15] Durka P. J. Malinowska U. Zieleniewska M. O’Reilly C. Różański P. T. Żygierewicz J. (2015). Spindles in svarog: framework and software for parametrization of EEG transients. Front. Hum. Neurosci. 9:258. 10.3389/fnhum.2015.00258 26005412 PMC4424848

[B16] Erdamar A. Duman F. Yetkin C. S. (2012). A wavelet and teager energy operator based method for automatic detection of K-complex in sleep EEG. Expert Syst. Appl. 39, 1284–1290. 10.1016/j.eswa.2011.07.138

[B17] Ferrarelli F. Peterson M. J. Sarasso S. Riedner B. A. Murphy M. J. Benca R. M.. (2010). Thalamic dysfunction in schizophrenia suggested by whole-night deficits in slow and fast spindles. Am. J. Psychiatry 167, 1339–1348. 10.1176/appi.ajp.2010.09121731 20843876 PMC2970761

[B18] Ferrarelli F. Tononi G. (2011). The thalamic reticular nucleus and schizophrenia. Schizophr. Bull. 37, 306–315. 10.1093/schbul/sbQ152 21131368 PMC3044616

[B19] Fogel S. Martin N. Lafortune M. Barakat M. Debas K. Laventure S.. (2012). NREM sleep oscillations and brain plasticity in aging. Front. Neurol. 3:176. 10.3389/fneur.2012.00176 23248614 PMC3522106

[B20] Fuentealba P. Steriade M. (2005). The reticular nucleus revisited: intrinsic and network properties of a thalamic pacemaker. Prog. Neurobiol. 75, 125–141. 10.1016/j.pneurobio.2005.01.002 15784303

[B21] Günes S. Dursun M. Polat K. Yosunkaya S. (2011). Sleep spindles recognition system based on time and frequency domain features. Expert Syst. Appl. 38, 2455–2461. 10.1016/j.eswa.2010.08.034

[B22] Halász P. (2005). K-complex, a reactive EEG grapho-element of NREM sleep: an old chap in a new garment. Sleep Med. Rev. 9, 391–412. 10.1016/j.smrv.2005.04.003 16122950

[B23] Huupponen E. Gómez-Herrero G. Saastamoinen A. Värri A. Hasan J. Himanen S. (2007). Development and comparison of four sleep spindle detection methods. Artif. Intell. Med. 40, 157–170. 10.1016/j.artmed.2007.04.003 17555950

[B24] Huupponen E. Värri A. Himanen S. L. Hasan J. Lehtokangas M. Saarinen J. (2000). Optimization of sigma amplitude threshold in sleep spindle detection. J. Sleep Res. 9, 327–334. 10.1046/j.1365-2869.2000.00220.x 11386202

[B25] Iber C. Ancoli-Israel S. Chesson A. L. Jr. Quan S. F. (2007). The AASM Manual for the Scoring of Sleep and Associated Events: Rules, Terminology and Technical Specification. Darien: American Academy of Sleep Medicine.

[B26] Jahnke K. von Wegner F. Morzelewski A. Borisov S. Maischein M. Steinmetz H.. (2012). To wake or not to wake? The two-sided nature of the human K-complex. Neuroimage 59, 1631–1638. 10.1016/j.neuroimage.2011.09.013 21945697

[B27] Jaleel A. Tafreshi R. Ahmed B. Boivin D. B. (2013). “Pilot validation of a mimicking algorithm for detection of sleep spindles and K-complexes,” in World Congresson Medical Physics and Biomedical Engineering May 26–31, 2012, Beijing, China, ed. Long M. (Berlin, Heidelberg: Springer Berlin Heidelberg), 562–565.

[B28] Jobert M. Poiseau E. Jähnig P. Schulz H. Kubicki S. (1992). Pattern recognition by matched filtering: an analysis of sleep spindle and K-complex density under the influence of lormetazepam and zopiclone. Neuropsychobiology 26, 100–107. 10.1159/000118902 1361968

[B29] Koley B. L. Dey D. (2012). “Detection of characteristic waves of sleep EEG by continuous wavelet transform,” in National Conference on Computing and Communication Systems (NCCCS) IEEE, 1–5, India.

[B30] Ktonas P. Y. Ventouras E.-C. (2014). Automated detection of sleep spindles in the scalp EEG and estimation of their intracranial current sources: comments on techniques and on related experimental and clinical studies. Front. Hum. Neurosci. 8:998. 10.3389/fnhum.2014.00998 25540616 PMC4261733

[B31] Lafortune M. Gagnon J.-F. Martin N. Latreille V. Dubé J. Bouchard M.. (2014). Sleep spindles and rapid eye movement sleep as predictors of next morning cognitive performance in healthy middle-aged and older participants. J. Sleep Res. 23, 159–167. 10.1111/jsr.12108 24245769

[B32] Lajnef T. Chaibi S. Eichenlaub J. B. Ruby P. M. Aguera P.-E. Samet M.. (2015a). Sleep spindle and K-complex detection using tunable Q-factor wavelet transform and morphological component analysis. Front. Hum. Neurosci. 9:414. 10.3389/fnhum.2015.00414 26283943 PMC4516876

[B33] Lajnef T. Chaibi S. Ruby P. Aguera P.-E. Eichenlaub J.-B. Samet M.. (2015b). Learning machines and sleeping brains: automatic sleep stage classification using decision-tree multi-class support vector machines. J. Neurosci. Methods 250, 94–105. 10.1016/j.jneumeth.2015.01.022 25629798

[B34] Latreille V. Carrier J. Lafortune M. Postuma R. B. Bertrand J.-A. Panisset M.. (2015). Sleep spindles in Parkinson’s disease may predict the development of dementia. Neurobiol. Aging 36, 1083–1090. 10.1016/j.neurobiolaging.2014.09.009 25442116

[B35] Lüthi A. (2014). Sleep spindles where they come from, what they do. Neuroscientist 20, 243–256. 10.1177/1073858413500854 23981852

[B36] Martin N. Lafortune M. Godbout J. Barakat M. Robillard R. Poirier G.. (2013). Topography of age-related changes in sleep spindles. Neurobiol. Aging 34, 468–476. 10.1016/j.neurobiolaging.2012.05.020 22809452

[B37] Mölle M. Marshall L. Gais S. Born J. (2002). Grouping of spindle activity during slow oscillations in human non-rapid eye movement sleep. J. Neurosci. 22, 10941–10947. 12486189 10.1523/JNEUROSCI.22-24-10941.2002 PMC6758415

[B38] Morin A. Doyon J. Dostie V. Barakat M. Hadj Tahar A. Korman M.. (2008). Motor sequence learning increases sleep spindles and fast frequencies in post-training sleep. Sleep 31, 1149–1156. 18714787 PMC2542961

[B42] O’Reilly C. Godin I. Montplaisir J. Nielsen T. (2015). REM sleep behaviour disorder is associated with lower fast and higher slow sleep spindle densities. J. Sleep Res. 24, 593–601. 10.1111/jsr.12309 26041532

[B43] O’Reilly C. Gosselin N. Carrier J. Nielsen T. (2014). Montreal archive of sleep studies: an open-access resource for instrument benchmarking and exploratory research. J. Sleep Res. 23, 628–635. 10.1111/jsr.12169 24909981

[B41] O’Reilly C. Nielsen T. (2015). Automatic sleep spindle detection: benchmarking with fine temporal resolution using open science tools. Front. Hum. Neurosci. 9:353. 10.3389/fnhum.2015.00353 26157375 PMC4478395

[B45] Parekh A. Selesnick I. W. Rapoport D. M. Ayappa I. (2015). Detection of K-complexes and sleep spindles (DETOKS) using sparse optimization. J. Neurosci. Methods 251, 37–46. 10.1016/j.jneumeth.2015.04.006 25956566

[B46] Patti C. R. Shahrbabaki S. S. Dissanayaka C. Cvetkovic D. (2015). “Application of random forest classifier for automatic sleep spindle detection,” in IEEE Biomedical Circuits and Systems Conference (BioCAS), 1–4, USA.

[B470] Ray L. B. Fogel S. M. Smith C. T. Peters K. R. (2010). Validating an automated sleep spindle detection algorithm using an individualized approach. J. Sleep Res. 19, 374–378. 10.1111/j.1365-2869.2009.00802.x 20149067

[B47] Ray L. B. Sockeel S. Soon M. Bore A. Myhr A. Stojanoski B.. (2015). Expert and crowd-sourced validation of an individualized sleep spindle detection method employing complex demodulation and individualized normalization. Front. Hum. Neurosci. 9:507. 10.3389/fnhum.2015.00507 26441604 PMC4585171

[B48] Rechtschaffen A. Kales A. (1968). A Manual of Standardized Terminology, Techniques and Scoring System for Sleep Stages of Human Subject. Washington, DC: Government Printing Office, National Institute of Health Publication.

[B49] Riemann D. Berger M. Voderholzer U. (2001). Sleep and depression—results from psychobiological studies: an overview. Biol. Psychol. 57, 67–103. 10.1016/s0301-0511(01)00090-4 11454435

[B50] Schabus M. Gruber G. Parapatics S. Sauter C. Klösch G. Anderer P.. (2004). Sleep spindles and their significance for declarative memory consolidation. Sleep 27, 1479–1485. 15683137 10.1093/sleep/27.7.1479

[B51] Schönwald S. V. de Santa-Helena E. L. Rossatto R. Chaves M. L. Gerhardt G. J. (2006). Benchmarking matching pursuit to find sleep spindles. J. Neurosci. Methods 156, 314–321. 10.1016/j.jneumeth.2006.01.026 16546262

[B52] Seeck-Hirschner M. Baier P. C. Weinhold S. L. Dittmar M. Heiermann S. Aldenhoff J. B.. (2012). Declarative memory performance is associated with the number of sleep spindles in elderly women. Am. J. Geriatr. Psychiatry 20, 782–788. 10.1097/jgp.0b013e31823033da 21997601

[B54] Selesnick I. W. (2011a). Wavelet transform with tunable Q-factor. IEEE Trans. Signal Process. 59, 3560–3575. 10.1109/TSP.2011.2143711

[B55] Selesnick I. W. (2011b). TQWT toolbox guide. Electrical and computer engineering, polytechnic institute of new york university. Available online at: http://eeweb.poly.edu/iselesni/TQWT/index.html

[B56] Selesnick I. W. (2011c). “Sparse signal representations using the tunable Q-factor wavelet transform,” in Proc. SPIE 8138, Wavelets and Sparsity XIV, 81381U, eds Papadakis M. Van De Ville D. Goyal V. K. (San Diego, CA:Wavelets and Sparsity XIV), 81381U.

[B53] Selesnick I. W. Bayram I. (2009). “Oscillatory and transient signal decomposition using over complete rational-dilation wavelet transforms, SPIE (society),” in Wavelets XIII: 2-4 August 2009, San Diego, California, United States, eds Goyal V. K. Papadakis M. Van De Ville D. (Bellingham: Wash SPIE), 74460V.

[B57] Steriade M. (2003). The cortico-thalamic system in sleep. Front. Biosci. 8, d878–d899. 10.2741/1043 12700074

[B58] Steriade M. (2005). Sleep, epilepsy and thalamic reticular inhibitory neurons. Trends Neurosci. 28, 317–324. 10.1016/j.tins.2005.03.007 15927688

[B59] Tsanas A. Clifford G. D. (2015). Stage-independent, single lead EEG sleep spindle detection using the continuous wavelet transform and local weighted smoothing. Front. Hum. Neurosci. 9:181. 10.3389/fnhum.2015.00181 25926784 PMC4396195

[B60] Warby C. S. Wendt S. L. Welinder P. Munk E. G. S. Carrillo O. Sorensen H. B. D.. (2014). Sleep-spindle detection: crowdsourcing and evaluating performance of experts, non-experts and automated methods. Nat. Methods 11, 385–392. 10.1038/nmeth.2855 24562424 PMC3972193

[B61] Zygierewicz J. Blinowska K. J. Durka P. J. Szelenberger W. Niemcewicz S. Androsiuk W.. (1999). High resolution study of sleep spindles. Clin. Neurophysiol. 110, 2136–2147. 10.1016/s1388-2457(99)00175-3 10616119

